# Melanoma Detection Using XGB Classifier Combined with Feature Extraction and K-Means SMOTE Techniques

**DOI:** 10.3390/diagnostics12071747

**Published:** 2022-07-19

**Authors:** Chih-Chi Chang, Yu-Zhen Li, Hui-Ching Wu, Ming-Hseng Tseng

**Affiliations:** 1 Department of Medical Informatics, Chung Shan Medical University, Taichung 402, Taiwan; s0858016@gm.csmu.edu.tw (C.-C.C.); s0858001@gm.csmu.edu.tw (Y.-Z.L.); 2 Department of Medical Sociology and Social Work, Chung Shan Medical University, Taichung 402, Taiwan; 3 Information Technology Office, Chung Shan Medical University Hospital, Taichung 402, Taiwan

**Keywords:** melanoma, feature extraction, transfer learning, imbalanced data, oversampling techniques, machine learning

## Abstract

Melanoma, a very severe form of skin cancer, spreads quickly and has a high mortality rate if not treated early. Recently, machine learning, deep learning, and other related technologies have been successfully applied to computer-aided diagnostic tasks of skin lesions. However, some issues in terms of image feature extraction and imbalanced data need to be addressed. Based on a method for manually annotating image features by dermatologists, we developed a melanoma detection model with four improvement strategies, including applying the transfer learning technique to automatically extract image features, adding gender and age metadata, using an oversampling technique for imbalanced data, and comparing machine learning algorithms. According to the experimental results, the improved strategies proposed in this study have statistically significant performance improvement effects. In particular, our proposed ensemble model can outperform previous related models.

## 1. Introduction

Malignant melanoma (MM) is the most severe form of skin cancer; although rare, it has a high mortality rate. According to GLOBOCAN statistics, in 2020, there were approximately 325,000 cases of melanoma skin-cancers worldwide, and melanoma accounted for 1.7% of the all-sites global cancer diagnoses. The calculations for the global age-standardized incidence rates show that the rate is 3.8/100,000 for males and 3.0/100,000 for females. The cumulative lifetime risk for males was 0.42% and for females was 0.33% [1]. MM easily spreads throughout the body, causing other cancers, such as brain, liver, and kidney cancers. Once it has spread, the survival rate will be less than 50%. The 5-year survival rate of patients is as high as 90 to 99%, if discovered early and resected; however, if detected late, the survival rate drops to approximately 15 to 20%. The actual cause of MM is unclear. At present, the medical community recognizes that exposure to ultraviolet light is a risk factor for the cancer. The frequency of MM in Asians is minimal, although it is more common on the palms/soles of the hands and feet, which are nonirradiated parts and hence unrelated to ultraviolet exposure. Recently, the incidence and mortality of MM have been increasing. Mortality caused by the cancer is common in young age groups, unlike the other types of cancer. Furthermore, a delay in medical treatment worsens the prognosis, causing metastasis, and even death; therefore, early diagnosis and treatment are essential [2,3].

In clinical diagnosis, it is difficult for dermatologists to identify early MM from a mole. South Queensland, Australia, has the highest global MM frequency. Edith Cowan University in Australia developed a blood test for MM antibodies, with an accuracy of 79% [4]; however, there are still testing and time cost limitations. Dermoscopy is a paramount technique for the initial diagnosis of MM. Therefore, if the development of artificial intelligence (AI) models of computer-aided diagnosis (CAD) systems can help dermatologists interpret the dermoscopy images, it will help to reduce medical costs.

Machine learning (ML) classifiers have been employed for the automatic diagnosis methods of skin lesions. Before modeling, these classifiers are input a set of handcrafted image features, such as the skin lesions-related features that dermatologists pay attention to. Recently, in most computer vision tasks, deep learning (DL) convolutional neural networks (CNNs) can automatically extract high-level image features and significantly improve the classification performance. Therefore, CNN-based CAD systems have been recently used to detect various diseases [5,6].

According to the latest review paper [7], on the topic of using neural networks to detect melanoma, the relevant architectures published in 2018–2021 were classified into the following four techniques. 1. Using a convolutional neural network; 2. Using multiple convolutional neural networks; 3. Using a convolutional neural network combined with other classifiers; 4. Using other techniques, such as combining ABCDE rules with traditional machine learning algorithms.

To test the performance of the AI models on dermoscopy images, there are many researchers who use public databases (such as PH2, MED-NODE, and ISIC). This article reviewed a total of 25 recent articles on MM CAD, published between 2016 and 2022, and listed the lowest and highest of the six indicators for the evaluation of efficacy, as shown in Table 1.

For example, Warsi et al. [9] used a 3D color texture feature (CTF) and a multilayer neural network model for the binary classification of MM diseases by a total of 200 dermoscopy images in the PH2 dataset. Based on the holdout method, 70% of the images were used as the training set, 15% of the images were used as the validation set, and 15% of the images were used as the test set. Their results showed the best performance in the PH2 dataset and reached 97.5% accuracy (ACC), 98.1% sensitivity (SEN), and 93.84% specificity (SPE). Iqbal et al. [14] proposed a new deep convolutional neural network (DCNN) model with multiple filter sizes: classification of the skin lesions network (CSLNet) architecture. Through data pre-processing and the data augmentation of ISIC-17, ISIC-18, and ISIC-19 images, it achieved 96.4% AUC, 93.25% ACC, 93.25% SEN, 90.64% SPE, 93.97% precision (PRE), and 94.47% F1 in the ISIC 2017 dataset, using the 7:1:2 holdout method.

To evaluate the performance of the MM prediction models using an oversampling technique, Kalwa et al. [28] used 200 dermoscopy images for the MM binary classification. By combining image feature extraction (FE), SVM, and synthetic minority oversampling technique (SMOTE) methods, the AUC was increased from 0.720 to 0.850. Magalhaes et al. [29] used 287 infrared thermography skin images for MM binary classification. Using an ensemble model of image FE, random forest (RF), SVM, and SMOTE methods, the recall increased from 0.473 to 0.696.

The contributions of this study are listed as follows:

1. Dermoscopy images (2299) were used for MM CAD, a dermatologist handcrafted feature method was used as a comparison base, and four classification efficiency improvement strategies were proposed: (1) a comparison of different transfer learning techniques for automatic image FE; (2) the addition of the metadata of gender and age; (3) a comparison of the class balance of the training data with different oversampling techniques; and (4) a comparison of the classification performance of different ML algorithms. According to the experimental results, the four proposed strategies are statistically significant for MM detection;
2. We combined the DL and ML methods to automatically extract the features directly from the dermoscopy images and perform benign and MM diagnosis. The experimental results show that our proposed model combining metadata, K-means SMOTE, and an extreme gradient boosting (XGB) classifier can achieve higher classification and predictability than using only the MELA-CNN feature extractor.

## 2. Methods

### 2.1. MM Dataset

In this study, we integrated the ISIC Challenge 2018 (ISIC2018) and the ISIC Challenge 2019 (ISIC2019) datasets [30,31,32,33] for the binary classification of benign and MM. The ISIC2018 dataset contains five handcrafted features provided by dermatologists: pigment networks; negative networks; streaks; globules; and milia-like cysts. Meanwhile, the ISIC2019 dataset contains two pieces of basic patient data: age and gender. There are 2299 records in this dataset, including 1849 benign and 450 MM. Because of the imbalanced data, subsequent processing is performed using oversampling techniques.

### 2.2. FE Techniques

FE is a preprocessing procedure in data mining. To evaluate the impact of the dermatologist handcrafted features [30] and automatic DL FE [34] on the classification performance of an ML algorithm for predicting MM, we compared the following five FE techniques.

**Figure 1:**
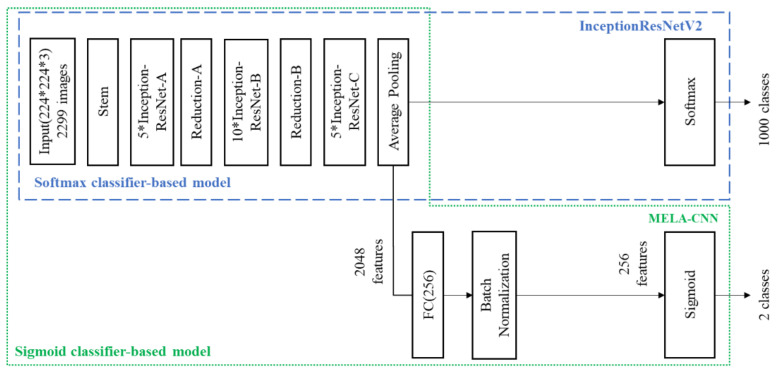
MELA-CNN network architecture.

(1) Handcraft: We employed five handcrafted characteristics provided by dermatologists [30]: pigment networks; negative networks; streaks; globules; and milia-like cysts. A pigment network is a grid comprising many brown lines crossing each other; a negative network is a curve formed by many hyperpigmented cell connections; a streak comprises pigmented projections surrounding a melanocytic lesion; a globule comprises multiple brown circles; a milia-like cyst comprises many white, yellowish circles or ovals;
(2) VGG16: VGG16 is a DL CNN model proposed by Karen Simonyan et al. [35]. They used the ImageNet dataset of one million images to classify one thousand classes. VGG16 takes 224 × 224 RGB images as the input and comprises 13 convolutional layers and 3 fully connected layers, as well as a nonlinear activation function—rectified linear unit (ReLU). All of the layers used three × three small convolution kernels, to avoid too many parameters. This DL model can automatically extract 512 features from the dermoscopy images;
(3) InceptionV3: InceptionV3 is a CNN-based DL model of the inception series. The inception series includes InceptionV1, InceptionV2, InceptionV3, InceptionV4, and InceptionResNet series. InceptionV3 was proposed by Szegedy et al. [36] as an improved InceptionV2. They used the ImageNet dataset of one million images to classify one thousand classes. InceptionV3 takes 224 × 224 RGB images as input and comprises 47 layers. In addition, this model adopts the batch normalization of InceptionV2 to accelerate the model training. This DL model can automatically extract 2048 features from dermoscopy images;
(4) InceptionResNetV2: InceptionResNetV2 is an Inception module-based DL model. It uses 299 × 299 RGB images as input. In addition, it replaces the pooling layers in the Inception modules A, B, and C, with ResNet connections to accelerate the training [37]. This DL model can automatically extract 1536 features from dermoscopy images;
(5) MELA-CNN: Based on the transfer learning technique [34], we used the InceptionResNetV2 architecture as the backbone to develop MELA-CNN (Figure 1). After retrieving the feature maps of the average pooling layer of InceptionResNetV2, a fully connected layer of 256 nodes is added, and ReLU is used. Further, batch normalization and Sigmoid layers are introduced, and MELA-CNN trained weights are obtained after the fine-tuning process using the target dataset. This DL model can automatically extract 256 features from dermoscopy images.

### 2.3. SMOTE

Because our datasets are from the medical field, the feature of a considerable numerical imbalance in the number of negative and positive samples is common. Therefore, we employed a data oversampling method to solve the imbalance in the number of data categories to avoid misjudgment of the classifier during training. Chawla et al. [38] proposed SMOTE, which randomly selects the k-nearest neighbor samples to increase the number of transactions in minority categories to the same number as the number of transactions in the majority category, to solve the problem of data imbalance. Because the SMOTE sampling technique is prone to generate noise and affect the classifier prediction performance, Douzas et al. [39] proposed K-means SMOTE, which is based on SMOTE and k-means clustering, for data oversampling. First, the data are grouped using the k-means method, and the clusters with minority classes accounting for less than 50% are selected. Then, the number of samples to be generated is calculated, and more samples are assigned to the clusters with sparse samples. Finally, SMOTE is performed in this cluster, and the number of minority samples is increased to the same number as the majority samples, solving the problem of data imbalance, and improving the shortcoming that SMOTE is prone to noise.

### 2.4. XGB

XGB, proposed by Tianqi Chen et al. [40], is based on the concept of gradient boosting decision tree (GBDT). GBDT is a gradient boosting algorithm based on a decision tree. Gradient boosting is an ensemble learning model that mainly trains the multiple weak classifiers, assembling them into a stronger classifier. The goal is to minimize the loss function and increase the weight of the misclassified classes by computing negative gradients to improve the next iteration of the training.

Compared with GBDT, XGB adds a regularization method, to make the loss function smoother, reduce the model complexity, and avoid overfitting. In addition, an approximation algorithm is used to find the optimal solution for splits, optimize the gradient boosting, and increase the efficiency and scalability. Further, considering the processing of missing or sparse values, it can be designated as a specific branch to improve the efficiency of an algorithm. Finally, to accelerate the model operation, XGB also supports a parallel operation and an early stop. When the prediction result reaches the optimum, the tree can be stopped in advance to increase the training speed. XGB can also improve the model classification accuracy.

### 2.5. Evaluation Metrics

To evaluate the performance of the different models for binary classification, we employed the confusion matrix to calculate the true positive (*TP*), true negative (*TN*), false positive (*FP*), and false negative (*FN*), as well as deriving the following five evaluation indicators:

Accuracy (ACC): The proportion of correct diagnoses in all of the samples.

(1) $$\mathrm{ACC}=\frac{TP+TN}{TP+TN+FP+FN}$$

Precision (PRE): The proportion of individuals who are positive in the group diagnosed with the disease.

(2) $$\mathrm{PRE}=\frac{TP}{TP+FP}$$

Recall (REC): The proportion of positive diagnosis results that are true positive, which is also called the true positive rate (TPR).

(3) $$\mathrm{REC}=\frac{TP}{TP+FN}$$

F1-score: The harmonic mean of PRE and REC.

(4) $$\frac{2}{F_{1}}=\frac{1}{PRE}+\frac{1}{REC}$$

AUC: The AUC of TPR and FPR. FPR is the false positive rate, which refers to the proportion of false positives in the actual disease-free population.

(5) $$\mathrm{FPR}=\frac{FP}{TN+FP}$$

The higher the value of the above five indicators, the better the classification performance of the model. Because of the use of ACC and PRE to evaluate the class-imbalanced dataset, the model may be biased due to numerous FNs. In this study, we aimed to develop a model that can effectively detect patients with MM. Therefore, we used REC, F1-score, and AUC as the main evaluation criteria for the model performance.

### 2.6. Stratified K-Fold Validation

We employed a stratified K-fold method for the 10-fold stratified cross-validation, which is an improvement of the K-fold cross-validation method. The K-fold cross-validation method divides the data into mutually exclusive k groups of equal sizes, and then repeats the training and testing k times. Each time, one group is used as the test data, and the others are used as the training data to verify the accuracy. Finally, the average of k times the accuracy is used as the final accuracy. The innovation of the stratified K-fold method is that each fold is extracted according to the category ratio for training and testing. Because the method ensures that the proportion of two categories in each fold is equal to the original dataset, it is suitable for imbalanced data classification.

### 2.7. Paired T-Test

To evaluate whether the difference in the MM detection ability using the proposed enhancement strategy is statistically significant, we used the paired *t*-test to compare the predictive performance of the two models:

(6) $$p=\frac{\bar{d}}{S_{d}/\sqrt{n}}$$

where $\bar{d}$ denotes the mean of the difference between paired data; $S_{d}$ denotes the standard deviation of the difference between paired data; and n denotes the number of pairs of data. The null hypothesis is a 10-fold validated REC or F1-score mean difference of 0 between the two models. When p < 0.05, it means that there is a statistically significant difference in the classification performance between the two models.

## 3. Proposed Framework

**Figure 2:**
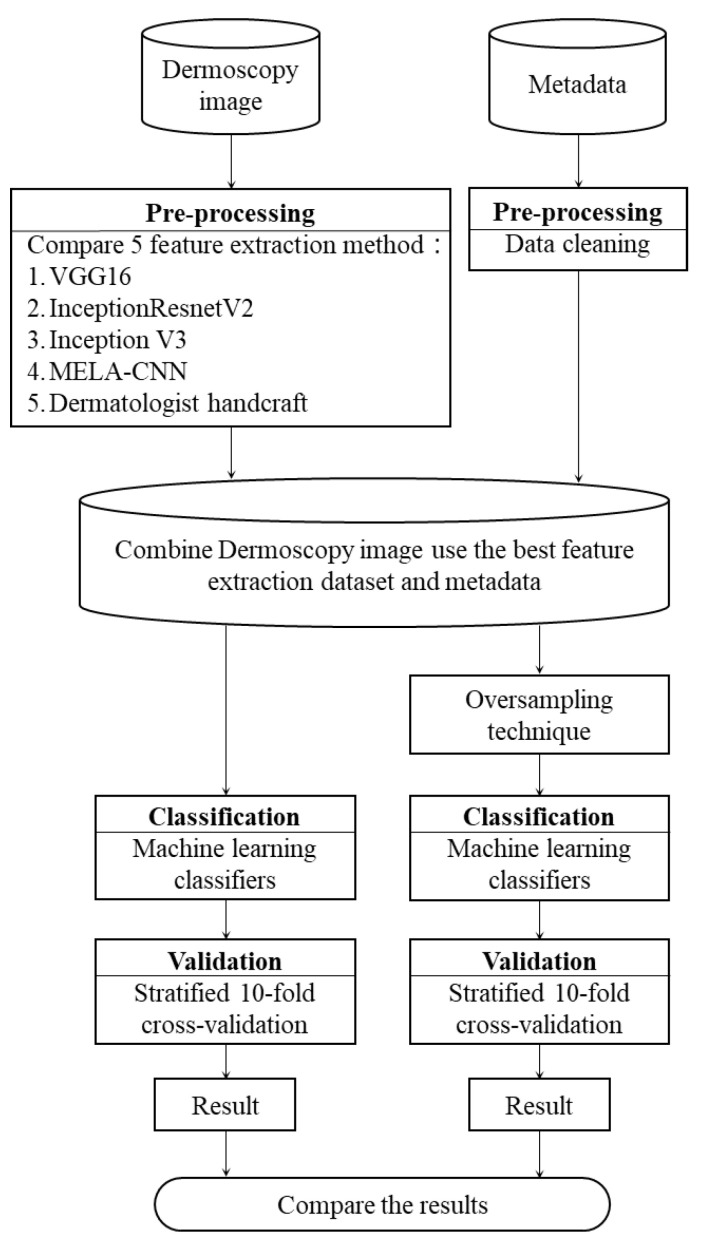
Research architecture.

In this study, we integrated the ISIC2018 dermoscopy image data [30,31] and the ISIC2019 patient age and gender basic data [32,33] to form a research dataset for developing an MM detection model. The overall research architecture is shown in Figure 2. First, the five FE methods were implemented on the dermoscopy images—VGG16, InceptionResNetV2, Inception V3, MELA-CNN, and the dermatologist handcrafted method. Then, we merged the optimal image features and metadata. Finally, we compared different oversampling techniques with different ML algorithms to find the optimal MM detection model.

**Figure 3:**
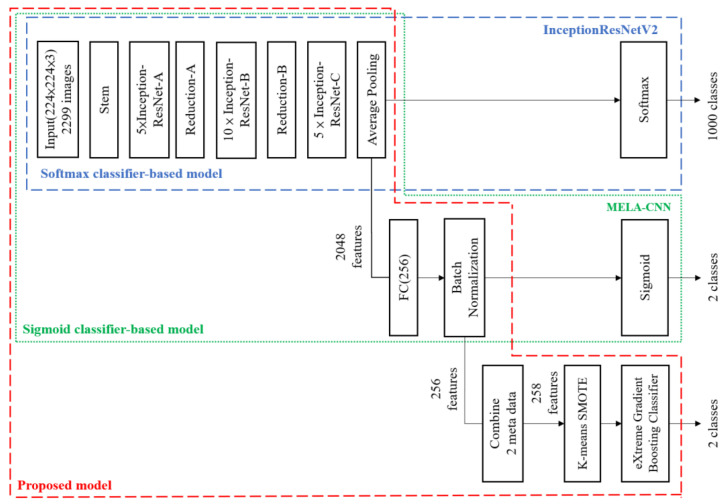
Proposed model architecture.

The proposed model architecture is shown in Figure 3. Based on the transfer learning technique [34], MELA-CNN is developed to automatically extract image features. In Figure 3, the first, second, and third block diagrams depict InceptionResNetV2, MELA-CNN, and the optimal MM detection model proposed in this study. The overall architecture of the proposed model is based on InceptionResNetV2 as the backbone and applies a fine-tuning process to train MELA-CNN for automatic image FE. Then, by combining two sets of metadata with the optimal image features, 258 features are obtained. In addition, we used K-means SMOTE for class balance. Finally, we employed XGB for MM detection.

## 4. Experimental Result

In this study, 2299 images were manually annotated by dermatologists in the ISIC2018 and ISIC2019 datasets to train and test the optimal classification model. In the process, the stratified K-fold method was used for 10-fold cross-validation, and data were extracted according to the proportion of categories. Then, we put them into each fold, performed 10 rounds of training and testing, and obtained the following results.

### 4.1. FE Techniques

Five techniques were used for the FE of dermoscopy images—the dermatologist handcrafted method, VGG16, InceptionResNetV2, Inception V3, and MELA-CNN—and the number of features after extraction was 512, 1536, 2048, and 256, respectively.

**Figure 4:**
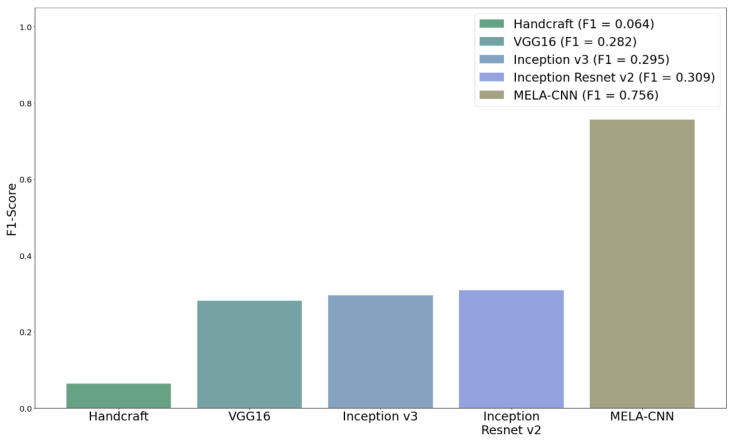
Comparison of F1-score of five feature extraction techniques.

Table 2 summarizes the results of the five techniques combined with XGB to compare their performance differences. Clearly, MELA-CNN was the most efficient method, with an F1-score value of 0.756. Meanwhile, the dermatologist handcrafted method had the worst performance, with an F1-score value of only 0.064. The F1-score values of VGG16, InceptionResNetV2, and Inception V3 are up to 0.282, 0.309, and 0.295, respectively. Figure 4 shows the performance comparison chart of the F1-score of the five FE techniques combined with XGB. Clearly, MELA-CNN significantly outperforms the other techniques.

### 4.2. Metadata

**Figure 5:**
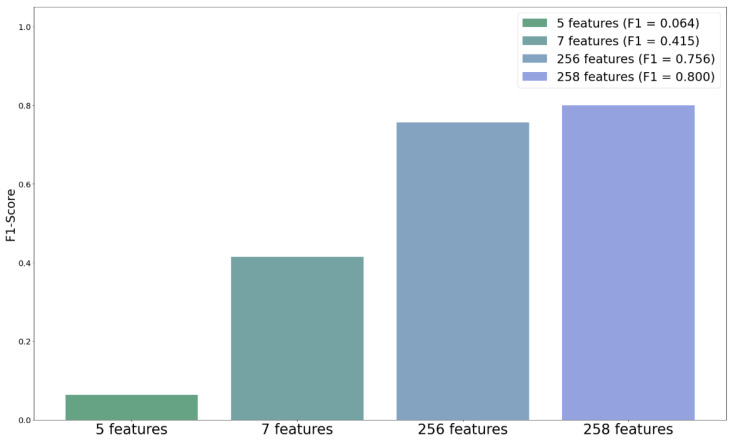
F1-score comparison of adding metadata.

To evaluate the dermoscopy image features by adding metadata, including age and gender, for the difference in the predictability of the diagnostic model, we employed XGB for the model training and used the F1-score as the main evaluation metric. The results in Table 3 show that the F1-score of the five symptoms obtained using the dermatologist handcrafted method, after adding metadata, can reach 0.415. Compared with the results without metadata, the F1-score increased by 35.1%. After adding metadata, the F1-score increased from 0.756 to 0.800, i.e., a 4.4% increase, for the 256 image features extracted by MELA-CNN. Figure 5 shows the F1-score performance comparison chart of 5 and 256 features with metadata. This figure clearly shows the relevance of the metadata. The classification performance of both of the models—the dermatologist handcrafted method and MELA-CNN—improved.

### 4.3. SMOTE

**Figure 6:**
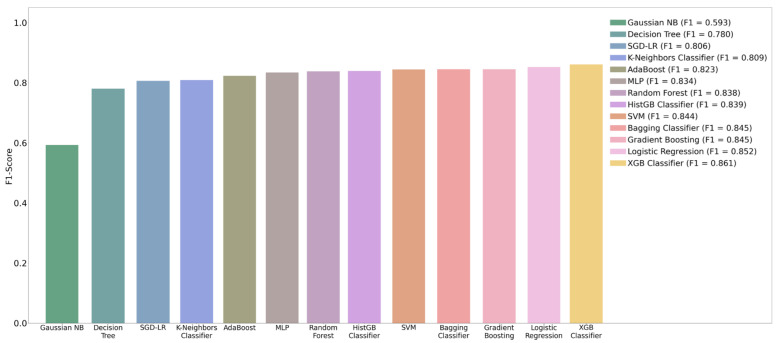
Comparison of F1-scores using 10 oversampling techniques.

Because of the problem of class imbalance in our datasets, we used 10 oversampling techniques to balance the classes of binary data with XGB, to assess the difference in performance. In this study, the oversampling technique was used only for the training set, and the test set was maintained in its original composition. The results in Table 4 represent the difference in performance on the test set that compared 10 oversampling techniques with the original no-sampling technique for model training. The results show that the original F1-score was only 0.800 without an oversampling technique. After using K-means SMOTE, which is the optimal oversampling method, the F1-score reached 0.861. The F1-score values of the other oversampling techniques are as follows. Random Over Sampler: 0.840; SMOTE: 0.839; SVMSMOTE: 0.835; SMOTETomek: 0.835; BorderlineSMOTE: 0.834; SMOTE-RandomUnderSampler: 0.831; SMOTENC: 0.830; SMOTEENN: 0.822; ADASYN: 0.814. Figure 6 shows the performance comparison chart of the F1-score under the 258 features obtained using the 10 oversampling techniques. Clearly, K-means SMOTE shows the most obvious improvement in the test performance of the original imbalanced training dataset.

### 4.4. ML Algorithms (Classifiers)

**Figure 7:**
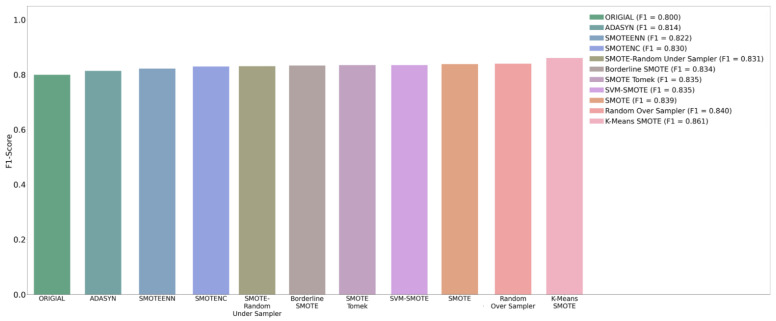
Comparison of F1-score of 13 classifiers with K-means SMOTE.

In this study, we compared the performance of 13 ML algorithms for MM classification, using the optimal results obtained using K-means SMOTE: XGB classifier, histogram-based gradient boosting (HistGB classifier), SVM, gradient boosting, RF, multilayer perceptron (MLP), Gaussian naive Bayes (Gaussian NB), logistic regression, bagging classifier, stochastic gradient descent logistic regression (SGD-LR), adaptive boosting (AdaBoost), decision tree, and K-neighbors classifier. Table 5 summarizes the results of the 13 ML algorithms for MM diagnosis. The F1-score of XGB is 0.861, which is the optimal classification performance. Figure 7 shows the F1-score performance comparison chart of the algorithms. Clearly, XGB significantly outperforms all of the other ML algorithms.

## 5. Discussion

### 5.1. Effect of FE and Metadata

**Figure 8:**
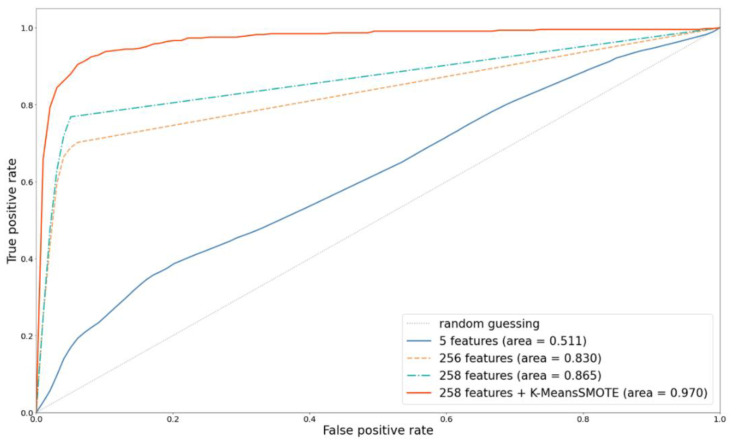
Comparison of ROC curves with different feature extractors.

**Figure 9:**
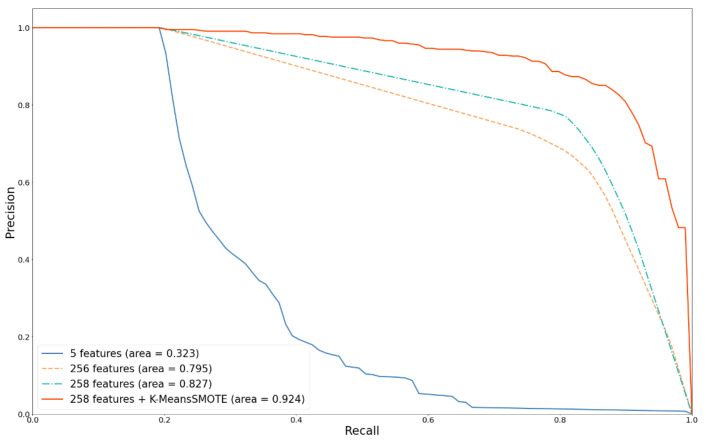
Comparison of PR curves with different feature extractors.

In this study, the dermatologist handcrafted method was used as a comparison basis to discuss the differences in the improvement of the performance for MM detection by four strategies: (1) using the automatic image FE method; (2) adding metadata; (3) using SMOTE; and (4) using different ML algorithms. Figure 8 and Figure 9 show the ROC and PRE–REC (PR) curves for four comparison FE techniques.

MELA-CNN is an automatic image FE technique; it could achieve the optimal classification performance because it performed the fine-tuning process on the target dataset. Compared with the handcrafted method, MELA-CNN has 31.9% and 47.2% increases in the AUC and the PR curve area, respectively. The results show that using MELA-CNN improves the predictability of the model compared with the handcrafted method.

Moreover, adding the metadata with 256 features could further improve the predictability of the model. The AUC and PR curve area increased to 0.865 and 0.827, respectively. Age and gender had a good resolution in the MM diagnosis. Finally, K-means SMOTE was used to tackle the problem of prediction bias, due to the imbalance of the data categories, and the AUC improved by as much as 0.970. These results once again show that the four proposed improvement strategies can effectively improve MM predictability.

### 5.2. Effect of Oversampling Techniques

**Figure 10:**
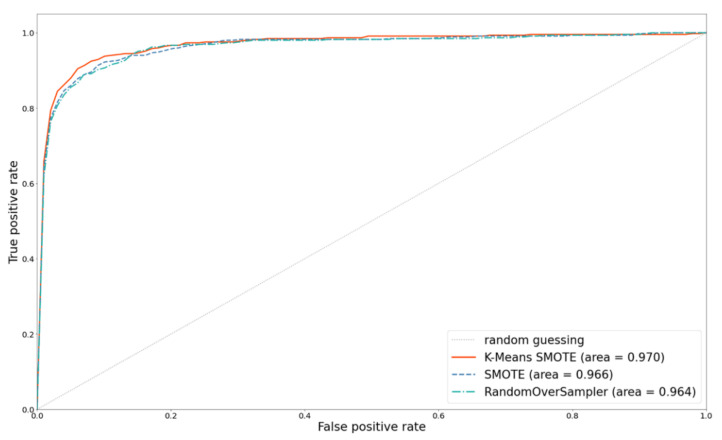
ROC curves comparison with different oversampling techniques.

**Figure 11:**
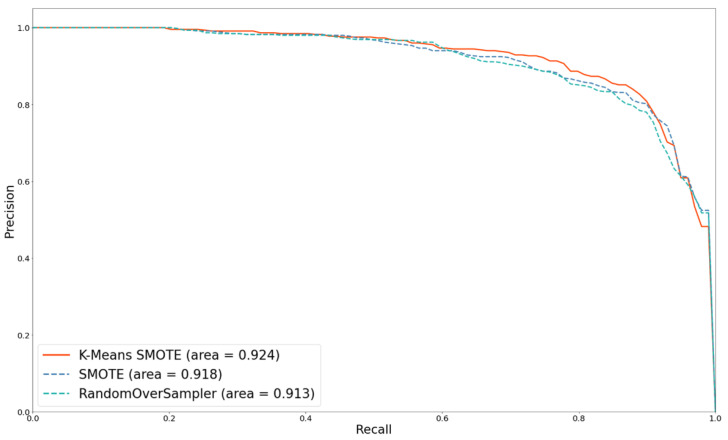
PR curves comparison with different oversampling techniques.

As mentioned above, using MELA-CNN for FE and adding the metadata with 258 features can improve the prediction performance. Therefore, this method is used as the basis, and K-means SMOTE, SMOTE, and RandomOverSampler are used as representative methods to compare the impact of the different oversampling techniques on classification performance. Compared with SMOTE, which randomly selects the k-nearest neighbor samples for oversampling without grouping, K-means SMOTE is a method for oversampling samples with denser minority classes in clusters. Using K-means SMOTE yields better results; its AUC and PR curve area can reach 0.970 and 0.924, respectively. Figure 10 and Figure 11 depict the ROC and PR curves for the performance comparisons of three oversampling methods. The results show that K-means SMOTE has the best classification and discernibility.

### 5.3. Effect of ML Algorithms (Classifiers)

**Figure 12:**
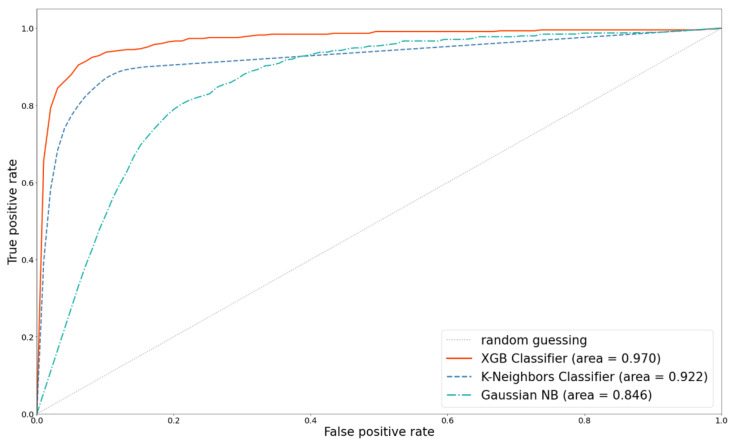
Comparison of ROC curves with different classifiers.

**Figure 13:**
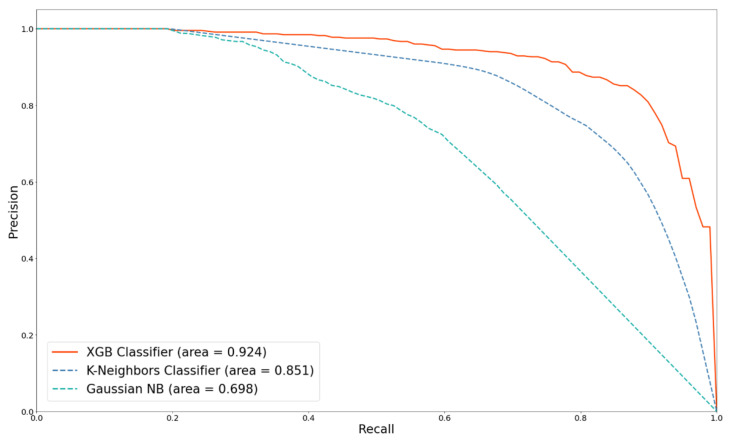
PR curves comparison with different classifiers.

As mentioned above, using 258 features with K-means SMOTE technology can achieve an optimal performance. Based on K-means SMOTE, we compared the differences in the prediction performance of different classifiers. Figure 12 and Figure 13 depict the ROC and PR curves for the performance comparison of three classifiers. Clearly, XGB has the best predictability and discernibility.

XGB, an ensemble learning classifier that combines multiple ML techniques, uses boosting to continuously train and revise weak learners to improve the prediction performance. It uses non-replacement random sampling to generate the different training subsets from the original training dataset and votes or averages for each training result to make the final prediction. Compared with the Gaussian NB and K-neighbors classifier, XGB has the best performance, with an AUC of 0.970 and a PR curve area of 0.924.

### 5.4. Significance Test for Performance Improvement

To assess whether the four improvement strategies proposed in this study are statistically significant, we employed XGB with the stratified 10-fold cross-validation method for five handcrafted features provided by dermatologists, 256 extracted features by MELA-CNN, 258 features obtained after adding the metadata of age and gender, and 258 features obtained by K-means SMOTE.

Table 6, Table 7 and Table 8 summarize the REC paired *t*-test results for the number of features of 5 versus 256, 256 versus 258, and 258 versus 258+K-means SMOTE, respectively. Besides, Table 9, Table 10 and Table 11 lists the corresponding F1-score paired *t*-test results. The null hypothesis is that the difference in 10-fold REC or F1-score between two models is 0. From Table 6, Table 7, Table 8, Table 9, Table 10 and Table 11, a significant *p*-value of less than 0.05 was obtained for all of the test results, thereby confirming the following three findings. (1) The use of the MELA-CNN feature extractor has a significant performance improvement over the dermatologist handcrafted method; (2) After adding the metadata of age and gender, the improvement in classification performance was also statistically significant; (3) Finally, there is also a statistically significant improvement in the predictive power of the model using K-means SMOTE.

### 5.5. Performance Comparison with Previous Related Studies

We compare five of the evaluation metrics of our research results and two previous studies [28,29] (as shown in Table 12 and Table 13). Based on the 7:3 holdout method, Kalwa et al. [28] performed only the binary MM classification for 200 dermoscopy images, combined with image FE, SVM, and SMOTE, the AUC can increase from 0.720 to 0.850. In this study, the binary MM classification was performed on 2299 dermoscopy images, and the proposed model was constructed by combining MELA-CNN, metadata, K-means SMOTE, and XGB and compared with five handcrafted image features provided by dermatologists, 1536 features extracted by the traditional transfer learning, 256 features extracted by MELA-CNN, and 258 by adding metadata. As a result, the AUC increased from 0.585 to 0.971, the F1-score increased from 0.056 to 0.890, and the REC increased from 0.030 to 0.867. Using the 8:2 holdout method, Magalhaes et al. [29] performed a binary classification of MM for 287 infrared thermography images. After combining image FE and the integrated model of RF and SVM with SMOTE, the REC can increase from 0.473 to 0.696. In this study, the AUC increased from 0.621 to 0.981, the F1-score increased from 0.063 to 0.905, and the REC increased from 0.033 to 0.878. The results in Table 12 and Table 13 confirm that the proposed model can obtain better classification and predictability than previous related models.

We also compared the performance of our proposed model with some other work in the literature. Based on the dataset, the imbalanced ratio (IR) of Non-Me and Me (IR) samples, the classification method, the validation method, and the performance of the test set, a comparative summary of these techniques is provided in Table 14. Since the different studies use different datasets and performance metrics, valid comparisons are difficult. However, the method proposed in this study still exhibits excellent performance.

## 6. Conclusions

Recently, the incidence of skin cancer has increased globally. The accurate classification of skin lesions directly influences the accurate and prompt diagnosis of skin cancer. MM is a highly lethal skin cancer that can rapidly metastasize, and eventually cause death if not detected early and treated properly.

Based on the method of expert manual annotation, an AI model for CAD of MM was developed for 2299 dermoscopy images in this study. We proposed four improvement strategies: (1) comparing different transfer learning techniques for automatic image FE; (2) adding the metadata of gender and age; (3) comparing different oversampling techniques for the class balancing of training data; and (4) comparing the classification performance of different ML algorithms. According to the experimental results, the proposed improvement strategies have a statistically significant effect on performance improvement.

After the analysis and comparison of the experimental results, we showed an effective combination of DL and ML methods to automatically extract features from dermoscopy images and perform benign and MM diagnoses. The experimental results also show that the proposed model, using the MELA-CNN feature extractor plus metadata, combined with K-means SMOTE and XGB, can obtain a better classification and prediction ability than the previous related models. Both the statistics and tests performed in this study confirmed that the proposed MM detection model has excellent classification performance.

However, if future clinical applications are to be met, it is necessary to further test the detection capabilities of a larger amount of case data and more categories of skin lesions to optimize the AI model. Based on the method proposed in this study, developing a computer-aided diagnosis system for melanoma with a user-friendly interface to support the clinical practice of dermatologists and provide an interpretation mechanism after automatic diagnosis is also the goal of the next stage of this study.

## Figures and Tables

**Table 1:** Performance evaluation test results on the models’ melanoma binary classification.

Authors	Dataset	AUC	ACC	SEN	SPE	PRE	F1
[8,9,10]	PH^2^	NA	0.861~0.975	0.790~0.981	0.925~0.938	NA	NA
[11]	Subset of PH^2^	NA	0.950	0.925	0.966	NA	NA
[12]	ISIC 2016	0.766	0.818	0.818	0.714	NA	0.826
[12,13,14]	ISIC 2017	0.870~0.964	0.857~0.933	0.490~0.933	0.872~0.961	0.940	0.813~0.935
[14,15,16,17,18,19,20,21]	ISIC 2018	0.847~0.989	0.803~0.931	0.484~0.888	0.957~0.978	0.860~0.905	0.491~0.891
[22,23]	Subset of ISIC 2018	0.970	0.880~0.910	0.920~0.960	NA	0.840~0.910	0.880~0.910
[14,20]	ISIC 2019	0.919~0.991	0.896~0.924	0.483~0.896	0.976~0.977	0.907	0.488~0.898
[11]	Subset of ISIC 2019	NA	0.930	0.925	0.933	NA	NA
[16,17,24,25]	Combined	0.880~0.960	0.803~0.950	0.851~0.930	0.844~0.950	NA	NA
[26]	MED-NODE	0.810	NA	0.810	0.800	NA	NA
[27]	Subset of ISBI 2017	0.891	0.866	0.556	0.785	NA	NA
NA = Metrics not mentioned in the paper							

**Table 2:** Performance evaluation of five feature extraction techniques.

Feature Extract	Features	ACC	PRE	REC	AUC	F1
Handcrafted	5	0.800	0.401	0.036	0.613	0.064
MELA-CNN	256	0.913	0.837	0.693	0.830	0.756
VGG16	512	0.814	0.569	0.189	0.738	0.282
InceptionResnet V2	1536	0.822	0.655	0.204	0.752	0.309
Inception V3	2048	0.819	0.641	0.198	0.746	0.295

**Table 3:** Performance evaluation of adding metadata.

Features	ACC	PRE	REC	AUC	F1
5	0.800	0.401	0.036	0.613	0.064
7	0.821	0.582	0.327	0.789	0.415
256	0.913	0.837	0.693	0.830	0.756
258	0.926	0.844	0.764	0.865	0.800

**Table 4:** Performance evaluation of 10 oversampling techniques.

Oversampling Technique	ACC	PRE	REC	AUC	F1
Original	0.926	0.844	0.764	0.864	0.800
K-Means SMOTE	0.946	0.873	0.853	0.970	0.861
RandomOverSampler	0.939	0.862	0.822	0.964	0.840
SMOTE	0.937	0.833	0.849	0.966	0.839
SVMSMOTE	0.934	0.825	0.851	0.967	0.835
SMOTETomek	0.934	0.829	0.844	0.967	0.835
BorderlineSMOTE	0.933	0.811	0.862	0.967	0.834
SMOTE- RandomUnderSampler	0.933	0.821	0.844	0.966	0.831
SMOTENC	0.932	0.820	0.849	0.968	0.830
SMOTEENN	0.924	0.770	0.889	0.967	0.822
ADASYN	0.924	0.788	0.847	0.966	0.814

**Table 5:** Performance evaluation of 13 classifiers with K-means SMOTE.

Classifiers	ACC	PRE	REC	AUC	F1
XGB Classifier	0.946	0.873	0.853	0.970	0.861
Logistic Regression	0.941	0.841	0.864	0.969	0.852
Gradient Boosting	0.940	0.851	0.842	0.965	0.845
Bagging Classifier	0.939	0.837	0.851	0.965	0.845
SVM	0.939	0.859	0.833	0.968	0.844
HistGB Classifier	0.939	0.861	0.822	0.968	0.839
Random Forest	0.936	0.837	0.842	0.964	0.838
MLP	0.937	0.862	0.811	0.963	0.834
AdaBoost	0.929	0.806	0.844	0.961	0.823
K-Neighbors Classifier	0.925	0.808	0.816	0.922	0.809
SGD-LR	0.922	0.783	0.836	0.956	0.806
Decision Tree	0.911	0.759	0.804	0.871	0.780
Gaussian NB	0.766	0.452	0.867	0.846	0.593

**Table 6:** Paired *t*-test of recall for 5 Features vs. 256 Features.

Fold	5 Features REC	256 Features REC	Difference between REC	Paired *t*-Test
1	0.022	0.578	0.556	p = 1.81 × 10^−9^ Average difference between REC 0.658
2	0.111	0.622	0.511	
3	0.044	0.756	0.712	
4	0.044	0.644	0.600	
5	0.044	0.800	0.756	
6	0.044	0.600	0.556	
7	0.000	0.733	0.733	
8	0.022	0.689	0.667	
9	0.000	0.756	0.756	
10	0.022	0.756	0.734	

**Table 7:** Paired *t*-test of recall for 256 features vs. 258 features.

Fold	256 Features REC	258 Feature REC	Difference between REC	Paired *t*-Test
1	0.578	0.844	0.267	p = 2.03 × 10^−2^ Average difference between REC 0.071
2	0.622	0.756	0.133	
3	0.756	0.778	0.022	
4	0.644	0.644	0.000	
5	0.800	0.778	−0.022	
6	0.600	0.756	0.156	
7	0.733	0.733	0.000	
8	0.689	0.778	0.089	
9	0.756	0.733	−0.022	
10	0.756	0.844	0.089	

**Table 8:** Paired *t*-test of recall for 258 features w/wo K-Means SMOTE.

Fold	258 Features REC	258 Features with K-Means SMOTE REC	Difference between REC	Paired *t*-Test
1	0.844	0.933	0.089	p = 7.07 × 10^−4^ Average difference between REC 0.089
2	0.756	0.867	0.111	
3	0.778	0.778	0.000	
4	0.644	0.844	0.200	
5	0.778	0.911	0.133	
6	0.756	0.889	0.133	
7	0.733	0.844	0.111	
8	0.778	0.800	0.022	
9	0.733	0.800	0.067	
10	0.844	0.867	0.022	

**Table 9:** Paired *t*-test of F1-score for 5 Features vs. 256 Features.

Fold	5 Features F1	256 Features F1	Difference between F1	Paired *t*-Test
1	0.042	0.658	0.616	p = 4.56 × 10^−10^ Average difference between F1 0.692
2	0.185	0.718	0.533	
3	0.083	0.810	0.727	
4	0.083	0.773	0.690	
5	0.083	0.818	0.735	
6	0.077	0.692	0.615	
7	0.000	0.767	0.767	
8	0.042	0.713	0.671	
9	0.000	0.810	0.810	
10	0.043	0.800	0.757	

**Table 10:** Paired *t*-test of F1-score for 256 features vs. 258 features.

Fold	256 Features F1	258 Features F1	Difference between F1	Paired *t*-Test
1	0.658	0.826	0.168	p = 3.40 × 10^−2^ Average difference between F1 0.040
2	0.718	0.791	0.073	
3	0.810	0.833	0.024	
4	0.773	0.734	−0.039	
5	0.818	0.795	−0.023	
6	0.692	0.810	0.117	
7	0.767	0.759	−0.009	
8	0.713	0.814	0.101	
9	0.810	0.815	0.005	
10	0.800	0.826	0.026	

**Table 11:** Paired *t*-test of F1-score for 258 features w/wo K-Means SMOTE.

Fold	258 Features F1	258 Features with K-Means SMOTE F1	Difference between F1	Paired *t*-Test
1	0.826	0.913	0.087	p = 3.35 × 10^−4^ Average difference between F1 0.061
2	0.791	0.813	0.022	
3	0.833	0.843	0.010	
4	0.734	0.874	0.139	
5	0.795	0.891	0.096	
6	0.810	0.870	0.060	
7	0.759	0.817	0.059	
8	0.814	0.857	0.043	
9	0.815	0.857	0.042	
10	0.826	0.876	0.050	

**Table 12:** Performance comparison with Kalwa et al. [28].

	Kalwa et al. (2019) [28]		Proposed Model				
	SVM (Kernel = RBF)		XGB Classifier				
	Holdout (7:3)		Holdout (7:3)				
	Original	SMOTE	Handcrafted	DL-TL	DL-FE	DL-FE+ Metadata	K-Means SMOTE
Number of samples	200		2299				
Number of features	4	4	5	1536	256	258	258
ACC	0.860	0.880	0.804	0.836	0.914	0.923	0.958
AUC	0.720	0.850	0.585	0.780	0.936	0.948	0.971
PRE	0.125	0.667	0.500	0.720	0.806	0.820	0.914
REC	0.500	0.800	0.030	0.267	0.741	0.778	0.867
F1	0.200	0.727	0.056	0.389	0.772	0.798	0.890

**Table 13:** Performance comparison with Magalhaes et al. [29].

	Magalhaes et al. (2021) [29]		Proposed Model				
	SVM + Random Forest		XGB Classifier				
	Holdout (8:2)		Holdout (8:2)				
	Original	SMOTE	Handcrafted	DL-TL	DL-FE	DL-FE+ Metadata	K-Means SMOTE
Number of samples	287		2299				
Number of features	40	40	5	1536	256	258	258
ACC	0.426	0.585	0.807	0.839	0.904	0.930	0.965
AUC	0.558	0.542	0.621	0.774	0.937	0.953	0.981
PRE	0.565	0.672	0.600	0.767	0.774	0.837	0.974
REC	0.473	0.696	0.033	0.256	0.722	0.800	0.878
F1	0.515	0.684	0.063	0.383	0.747	0.818	0.905

**Table 14:** A comparative summary of the existing techniques for melanoma binary classification.

Year	Author	Dataset	Non-Me: Me (IR)	Method	Validation	Test Result
2016	Nasr et al. [26]	MED-NODE	100:70 (1.429)	DL	Holdout (8:2) full: 7650	ACC: 0.810 SE: 0.810 SP: 0.800
2018	Adjed et al. [8]	PH^2^	160:40 (4)	Multiresolution technique + ML	Repeat 1000 times Holdout (7:3) full: 200	ACC: 0.861 SE: 0.790 SP: 0.933
2018	Li et al. [15]	ISIC 2018	8902:1113 (7.998)	DL + ML	Holdout (7:1:2) full: 10015	ACC: 0.853 PRE: 0.860 REC: 0.850 F1: 0.860
2019	Devansh et al. [41]	Combine of ISIC 2017, Edinburgh data, ISIC 2018, PH^2^	3063:919 (3.333)	DL	Holdout (85:15) full: 3982	AUC: 0.880
2019	Warsi et al. [10]	PH^2^	160:40 (4)	3D color-texture feature (CTF) + DL	Holdout (70:15:15) full: 200	ACC: 0.970 SE: 0.981 SP: 0.925
2019	Abbes et al. [24]	Combine of DermQuest and DermIS	87:119 (0.731)	FCM + DL	Holdout (NA) full: 206	ACC: 0.875 SE: 0.901 SP: 0.844
2019	Abbas et al. [25]	Subset of combining Skin-EDRA, ISIC 2018, DermNet, PH^2^	1420:1380 (1.029)	DL + ML	Holdout (1:1) full: 2800	ACC: 0.950 AUC: 0.960 SE: 0.930 SP: 0.950
2020	Almaraz-Damian et al. [19]	ISIC 2018	8902:1113 (7.998)	DL + ML	Holdout (75:25) full: 10015	ACC: 0.897
2020	Daghrir et al. [42]	Subset of ISIC archive	NA	DL+ML	Holdout (8:2) full: 640	ACC: 0.884
2022	Iftiaz A. Alf et al. [23]	Subset of ISIC 2018	1800:1497 (1.202)	DL and ML	Holdout (8:2) full: 3297	DL ACC: 0.910 PRE: 0.910 REC: 0.920 AUC: 0.970 F1: 0.910 ML ACC: 0.880 PRE: 0.840 REC: 0.920 F1: 0.880
2022	Our approach (Holdout 8:2)	Subset of combining ISIC 2018 and ISIC 2019	1849:450 (4.109)	DL + ML	Holdout (8:2) full: 2299	ACC: 0.965 PRE: 0.974 REC: 0.878 AUC: 0.981 F1: 0.905
2022	Our approach (Stratified 10-fold Cross Validation)	Subset of combining ISIC 2018 and ISIC 2019	1849:450 (4.109)	DL + ML	Stratified 10-fold Cross-Validation full: 2299	ACC: 0.941 PRE: 0.870 REC: 0.822 AUC: 0.968 F1: 0.844

## Data Availability

Not applicable.
